# Electromyographic Permutation Entropy Quantifies Diaphragmatic Denervation and Reinnervation

**DOI:** 10.1371/journal.pone.0115754

**Published:** 2014-12-22

**Authors:** Christopher Kramer, Denis Jordan, Alexander Kretschmer, Veronika Lehmeyer, Kristine Kellermann, Stephan J. Schaller, Manfred Blobner, Eberhard F. Kochs, Heidrun Fink

**Affiliations:** Klinik für Anaesthesiologie, Technische Universität München, Klinikum rechts der Isar, Ismaninger Str. 22, 81675, München, Germany; West Virginia University School of Medicine, United States of America

## Abstract

Spontaneous reinnervation after diaphragmatic paralysis due to trauma, surgery, tumors and spinal cord injuries is frequently observed. A possible explanation could be collateral reinnervation, since the diaphragm is commonly double-innervated by the (accessory) phrenic nerve. Permutation entropy (PeEn), a complexity measure for time series, may reflect a functional state of neuromuscular transmission by quantifying the complexity of interactions across neural and muscular networks. In an established rat model, electromyographic signals of the diaphragm after phrenicotomy were analyzed using PeEn quantifying denervation and reinnervation. Thirty-three anesthetized rats were unilaterally phrenicotomized. After 1, 3, 9, 27 and 81 days, diaphragmatic electromyographic PeEn was analyzed in vivo from sternal, mid-costal and crural areas of both hemidiaphragms. After euthanasia of the animals, both hemidiaphragms were dissected for fiber type evaluation. The electromyographic incidence of an accessory phrenic nerve was 76%. At day 1 after phrenicotomy, PeEn (normalized values) was significantly diminished in the sternal (median: 0.69; interquartile range: 0.66–0.75) and mid-costal area (0.68; 0.66–0.72) compared to the non-denervated side (0.84; 0.78–0.90) at threshold p<0.05. In the crural area, innervated by the accessory phrenic nerve, PeEn remained unchanged (0.79; 0.72–0.86). During reinnervation over 81 days, PeEn normalized in the mid-costal area (0.84; 0.77–0.86), whereas it remained reduced in the sternal area (0.77; 0.70–0.81). Fiber type grouping, a histological sign for reinnervation, was found in the mid-costal area in 20% after 27 days and in 80% after 81 days. Collateral reinnervation can restore diaphragm activity after phrenicotomy. Electromyographic PeEn represents a new, distinctive assessment characterizing intramuscular function following denervation and reinnervation.

## Introduction

Amongst all respiratory muscles, the diaphragm is the most important to ensure respiration. Uni- or bilateral diaphragmatic paralysis impairs breathing function and frequently results in respiratory failure. Besides idiopathic causes such as trauma, surgery, tumors, spinal cord injuries, neuromuscular disorders, chiropractic manipulations and infective neuropathies are the most common etiologic factors involved [1]–[4]. However, reinnervation can restore diaphragmatic function. In a study from Douglas and Calgett 6 of 40 patients with idiopathic unilateral diaphragmatic paralysis spontaneously recovered after 2 to 19 years [5]. In newborns, spontaneous reinnervation of unilateral diaphragmatic paralysis caused by phrenic nerve birth damage has been described in 5 of 23 patients within 1 month [6]. Main neural input to the diaphragm is provided by the phrenic nerve. However, parts of the crural regions, both in humans and rats, are innervated by the accessory phrenic nerve [7]–[8]. In humans, the accessory phrenic nerve is anatomically present in 53–61% of individuals and usually joins the main phrenic nerve anterior to the subclavian vein [9]–[10]. In rats, the accessory phrenic nerve has an incidence of 84%, derives from C6 and joins the phrenic nerve below the first rib [7]. After phrenicotomy only the mid-costal and sternal parts of the diaphragm are paralyzed, while innervation of the crural diaphragmatic region is maintained by the accessory phrenic nerve [8]. In case of phrenic nerve denervation it seems feasible that collateral reinnervation and therefore muscular reinnervation improving diaphragmatic muscular strength, can originate from the accessory phrenic nerve. In a patient, resection of a cervical accessory phrenic nerve Schwannoma resulted in a partial paralysis of the crural part of the respective hemidiaphragm. As a result of collateral reinnervation of the crural diaphragmatic fibers, a significant improvement of the vital capacity of the patient was observed after 6 months [11]. This rehabilitation potential of the double-innervated diaphragm was the basis of the here electrophysiologically studied effects of denervation and reinnervation thereafter.

One of the main electrophysiologic diagnostic tools to identify and characterize neuromuscular dysfunctions is electromyography [12] which has mostly been evaluated by amplitudes and frequency analysis. However, more sophisticated analysis methods seem to be more adequate for the quantitative assessment of muscular function. Especially non-linear entropy based approaches hold promise for quantification of the complexity of functional physiological processes induced by changes of underlying neuromuscular plasticity during denervation and reinnervation. The present investigation evaluates electromyographic permutation entropy (PeEn) to indicate denervation and reinnervation of the diaphragm. PeEn allows a reliable analysis of short and disturbed signals and it is expected to represent an expedient approach of electromyographic signals. Electromyographic quantification of denervation and reinnervation is of clinical interest, since denervation and reinnervation are etiologic for various chronic neuropathies, e.g. diabetes mellitus, Guillain-Barré-Syndrome or amyotrophic lateral sclerosis [13]–[14] in which electromyography is a main diagnostic tool. We hypothesize that reinnervation with forming larger motor units (fiber type grouping) by branching of the accessory phrenic nerve nerve axons to the denervated muscle fibers leads to a higher complexity of the neuromuscular network structure and therefore higher entropy values.

## Materials and Methods

This study was performed in strict accordance with the recommendations in the “Guide for the Care and Use of Laboratory Animals of the National Institutes of Health”. Governmental approval of the study was obtained (Regierung von Oberbayern, Munich, Germany *AZ 55.2-1-54-2531-115-08*). Thirty-three male Sprague Dawley rats (Charles River GmbH, Kisslegg, Germany, 280–330 g) were allowed to accommodate in our animal facility under a 12∶12-h light-dark cycle with free access to standard rat chow and water for at least 7 days. Body weight was measured daily. Relevant data are available at Dryad: doi:10.5061/dryad.p725k.

### Study design

The animals were randomly assigned to one of the four subgroups according to 1 (n = 6), 3 (n = 6), 9 (n = 6), 27 (n = 6) and 81 (n = 10) days after denervation. All rats randomly received either right or left cervical phrenicotomy (termed “denervated”) as previously described [15]. The contralateral side of the diaphragm was left intact and served as control, termed “non-denervated”. The central tendon of the diaphragm as a connective tissue sheet electrophysiologically separates both hemidiaphragms. This allows contralateral recordings to remain independent from recordings of the denervated side [4]. At the respective day after denervation, in vivo electromyographic activity of the diaphragm was recorded. Immediately afterwards the anesthetized rats were killed by exsanguination and specimens of both hemidiaphragms were obtained for histologic fiber type classification.

### Anesthesia and vital parameters

For the phrenicotomy operation, animals were anesthetized with isoflurane in a plexiglas chamber. Once anesthetized, the animals were endotracheally intubated and mechanically ventilated with oxygen in air (ratio 1∶2), while anesthesia was maintained with isoflurane (inspiratory fraction: 1.0 Vol%) and fentanyl i.v. (50 µg/kg via the crural penile vein). After surgery, animals were extubated and allowed to recover for 2 h before being returned to their cages.

For the electromyographic recordings, anesthesia at the respective day was also induced by inhalation of isoflurane in a plexiglas chamber. After loss of consciousness, the rats were endotracheally intubated while breathing spontaneously oxygen in air (ratio 1∶2). To preserve spontaneous breathing for the electromyographic recordings, anesthesia was maintained with 1.0 Vol% isoflurane. Rectal temperature was controlled between 36.8–37.2°C using a heating lamp. After electromyographic recordings, the animals were killed by exsanguination. Subsequently, both hemidiaphragms were excised, and one specimen each from the sternal, mid-costal and crural area was taken from both hemidiaphragms and rapidly frozen in isopentane precooled by liquid nitrogen for histologic analysis.

### Surgical procedure

Unilateral phrenicotomy was performed randomly either on the right or left side in the neck area. To prevent direct reinnervation, approximately 20 mm of the phrenic nerve were transected beneath the sternomastoid muscle and both, the proximal and distal nerve stump, were separated and cauterized as previously described [15]–[16]. During this procedure, the accessory phrenic nerve remained unaffected.

### Quantitative electromyography in vivo

For recording the bilateral electromyographic activity of the diaphragm, the rats were laparatomized and the diaphragm exposed. Pairs of monopolar platinum needle electrodes (0.76 mm diameter, model 515015, Schwarzer, Munich, Germany) were placed into the right and left hemidiaphragm.

**Figure 1:**
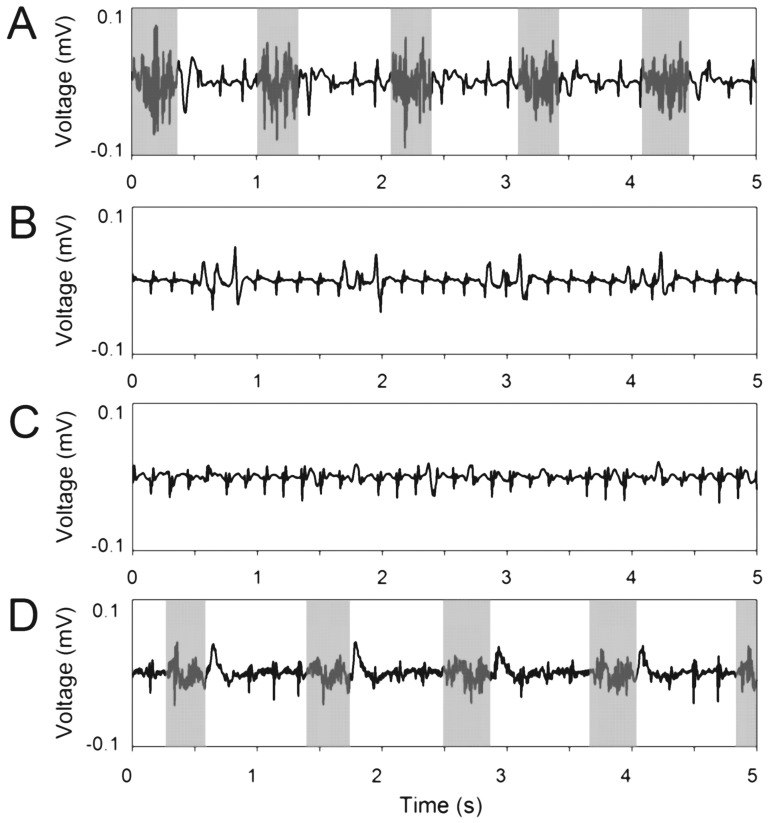
Spontaneous electromyographic activity of a non-denervated (A) and denervated sternal (B), mid-costal (C) and crural (D) hemidiaphragm 1 day after phrenicotomy. While the non-denervated hemidiaphragm shows characteristic, inspiration-related electromyographic bursts (gray areas; manually labelled), on the denervated side mainly electrocardiographic traces were observed. Burst epochs were manually identified and used for electromyographic analysis (A, B: grey intervals). Otherwise, if burst epochs were not clearly identifiable, the complete recording was included in analysis (B, C).

A four-channel electromyogram was recorded at a sampling rate of 44.1 kHz consecutively on the sternal, mid-costal and crural part of the denervated side and on the mid-costal part of the contralateral hemidiaphragm, the control, using differential amplifiers (AC/DC Strain Gage Amplifier, model P122 rackmount style, Grass Technologies, West Warwick, Rhode Island, USA). Voltage gain was adjusted for each recording to utilize the amplifiers dynamic range. Electromyographic activity was monitored via an oscilloscope (digital 4-channel oscilloscope, model DPO2014, Tektronix, Beaverton, Oregon, USA) and continuously recorded during spontaneous breathing for 20 seconds at a minimum (see Fig. 1).

### Non-linear Permutation Entropy

Permutation Entropy (PeEn) has been presented by Bandt et al. in 2002 as a measure of the irregularity of signals based on a comparison of the neighboring order of signal values [17]. PeEn was already introduced as an effective electroencephalographic measure indicating consciousness and unconsciousness during general anesthesia [18]–[20]. In the present analysis, PeEn was calculated for the characterization of electromyographic signals for quantitative assessment of reinnervation-induced irregularities. PeEn analyzes consecutive sequences 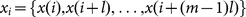 (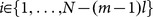) with respect to embedding parameters *m* (dimension) and *l* (time lag) in the analyzed signal (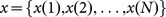) of length *N*. The order of samples in every sequence according to their amplitudes is computed and defines permutations of order *m*. PeEn is given by the Shannon entropy of the distribution of the obtained permutations and quantifies the monotone behavior of adjacent signal amplitudes. Therefore PeEn remains independent from absolute amplitude values (e.g. voltage gain in electromyographic recordings). A key advantage of the non-parametric PeEn over parametric analysis methods is its robustness against artifacts, signal distortions and poorly known characteristics of the underlying dynamics which makes this approach comparatively robust to a specific selection of embedding parameters (dimension *m*, time lag *l*). Furthermore it provides a reliable analysis of a short time series even if generated by high dimensional systems [19], [21]–[22]. Therefore, PeEn may represent an expedient method to quantify the complexity of the neuromuscular network structure and therefore to indicate effects of denervation and reinnervation in the electromyogram.

For electromyographic analysis, the frequency *f*_80_ = 173 Hz (mean value over all signals) splitting the spectral power at 80% below *f*_80_ and 20% above *f*_80_ was used to determine the high edge frequency. To reduce influence of the electrocardiogram as included in the electromyographic recordings a low edge frequency of 10 Hz was considered for analysis. This information was used to provide an appropriate frequency range (*f*_l_ to *f*_h_) and sampling frequency *f*_s_ for PeEn calculation.

Burst epochs used for electromyographic analysis were identified manually (Fig. 1A, D). If bursts were not clearly identifiable, the complete recording was analyzed (Fig. 1B, C). Primary analysis of PeEn was performed after band pass filtering between *f*_l_ = 10 Hz and *f*_h_ = 170 Hz (digital Butterworth filters of 3^rd^ order) and down sampling at *f*_s_ = 400 Hz. The embedding dimension *m* = 7 was chosen to reflect “high dimensionality” of the generating process, even if short burst epochs of around 500 ms may cause a bias at *m*>4 [19], [21] in estimation of PeEn. Time lag *l* = 3 provides a suitable deployment of trajectories (i.e. independency of consecutive samples) in the phase space [21].

A secondary analysis was performed to verify robustness of the results at different scales and frequency settings of PeEn. Therefore, PeEn was calculated after filtering with *f*_l_ = 0.5 Hz–20 Hz, *f*_h_ = 100 Hz–400 Hz (*f*_s_ = 400 Hz–1 kHz), *m* = 4–7 and *l* = 1–5.

For statistical analysis, one mean value of PeEn for each electromyographic recording was used. Signal processing was performed using LabView 6.0 (National Instruments, Austin, TX) on a Windows 7 platform (Microsoft Corporation, Redmond, WA).

### Fiber type classification

10 µm cryosections from both hemidiaphragms were obtained from each rat, sampling sternal, mid-costal and crural area of the diaphragm. After preincubation at pH 9.4 and 4.5, sections were enzyme-histochemically stained for myofibrillar ATPase [23]. Based on the staining pattern, muscle fibers were classified as type 1 ( = slow), 2a (fast), 2b or 2x (fast). Three visual fields in the respective area of each hemidiaphragm were examined for groups of the same muscle fiber type (fiber type groupings), a histological parameter for reinnervation (model AxioVision 3.1; Carl Zeiss, Oberkochen, Germany) [24].

### Statistical analyses

The electromyographic data reported are median and interquartile ranges of PeEn which was statistically analyzed with SPSS 11.0 (SPSS Inc., Chicago, IL) and R 2.15.2 (R Foundation, Vienna, Austria) on a Windows 7 platform. Effects of denervation and consecutive reinnervation were identified using a quantile regression (linear model based on median), where uncertainty of the slope parameter was estimated at threshold p<0.05 (Bonferroni correction). Differences of PeEn between day 1 vs. non-denervated side and day 1 vs. day 81 were indicated using a Mann-Whitney-U test at threshold p<0.05 (Bonferroni correction). The effect of time (day 1 to day 81) on PeEn within the non-denervated was evaluated using a Kruskal-Wallis test at threshold p<0.05.

Changes of fiber type grouping were evaluated for myofibrillar ATPase stainings and analyzed by aχ^2^-test at threshold p<0.05 (Fisher's exact test).

## Results

One animal of the 27 days group died 12 days after the phrenicotomy due to unknown reasons. Body weight increased continuously during the 81 days in all animals with no effect of denervation/reinnervation.

### Clinical observation of reinnervation

The non-denervated side showed typical rhythmic phases of contraction of the complete hemidiaphragm. A remaining contraction of the crural area of the denervated hemidiaphragm was visually observed in 4 of the 6 rats of the day 1, 3 and 9 group, respectively at the time of laparotomy. After 27 days, contractions of the phrenicotomized hemidiaphragms were seen in the crural and mid-costal regions in 4 of the 5 rats. After 81 days, 8 of 10 rats showed contractions of the initially denervated side with no visual difference to the non-denervated side.

### Histologic signs of reinnervation

**Figure 2:**
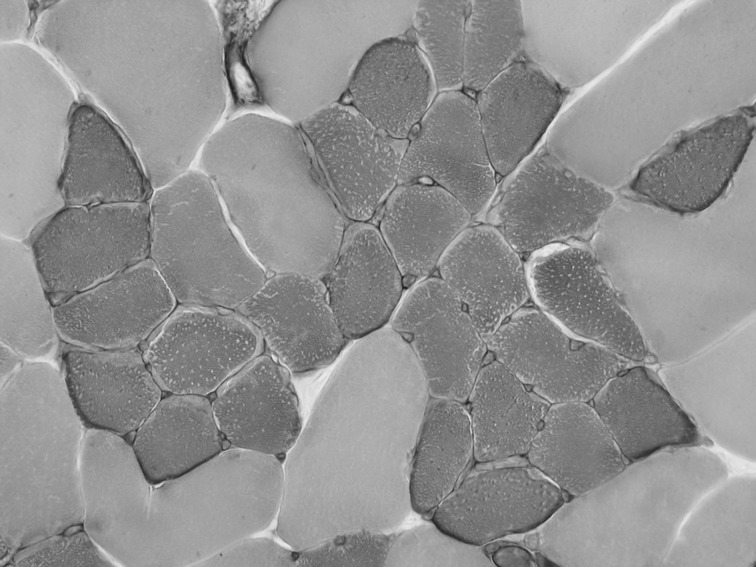
Exemplarily fiber type grouping in one rat as a histologic parameter of reinnervation (81 days after phrenicotomy in the mid-costal area).

Fig. 2 exemplarily shows a fiber type grouping in one rat as a sign of reinnervation (81 days after phrenicotomy in the mid-costal area). Fiber type grouping was observed in 20% of rats in the mid-costal and 40% in crural parts of the phrenicotomized hemidiaphragms after 27 days and in 80% and 90% after 81 days, respectively (Table 1). No fiber type grouping was observed in the sternal parts of the phrenicotomized hemidiaphragms and in the non-denervated hemidiaphragm.

**Table 1:** Fiber type grouping during reinnervation after 1, 3, 9, 27 and 81 days of phrenicotomy.

Fiber type grouping	Day 1	Day 3	Day 9	Day 27	Day 81	Non-denervated
Sternal	0%	0%	0%	0%	0%	0%
Mid-costal	0%	0%	0%	20%	80%* (p = 0.047)	0%
Crural	0%	0%	0%	40%	90% (p = 0.074)	0%

Percentage of subjects showing fiber type grouping in hemidiaphragms during reinnervation.

*: Significant difference in the mid-costal area at day 27 vs. day 81 (threshold p<0.05).

### Permutation Entropy

**Figure 3:**
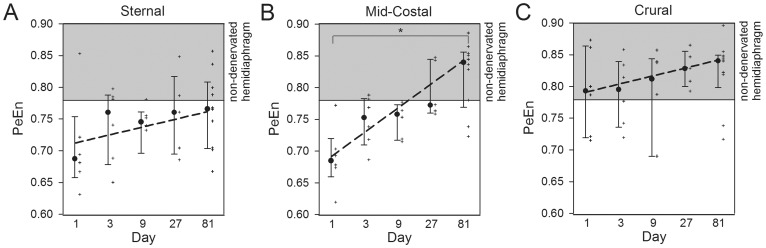
Median, interquartile ranges, values (+) and quantile regression (dashed line) of normalized permutation entropy (PeEn) values in the sternal (A), mid-costal (B) and crural (C) area 1–81 days after phrenicotomy such as in the non-denervated side (grey area: minimum and maximum). *: Significant increase of PeEn from day 1 to day 81 (quantile regression, threshold p<0.05).

After phrenicotomy, 25 of 33 rats (76%), showed PeEn values comparable to the non-denervated side in the crural hemidiaphragms indicating the presence of an accessory phrenic nerve (Fig. 3). As shown in Table 2 on day 1, PeEn was significantly reduced in the sternal and mid-costal hemidiaphragm compared to the non-denervated hemidiaphragm whereas PeEn of the crural area remained unchanged. While PeEn of the mid-costal area showed increased values over the time course of reinnervation until reaching the level of non-denervated side, PeEn of the sternal area remained decreased (Table 2 and Fig. 3).

**Table 2:** Normalized permutation entropy (PeEn) at the sternal, mid-costal and crural area 1, 3, 9, 27 and 81 days after phrenicotomy such as in the non-denervated side.

PeEn	Day 1	Day 3	Day 9	Day 27	Day 81	Day 1–81 regression	Non-denervated
						I: intercept, S: slope	(day 1–81)
Sternal	0.69; 0.66–0.75^#^ (p = 0.01)	0.76; 0.68–0.79	0.75; 0.69–0.76	0.76; 0.69–0.82	0.77; 0.70–0.81 (p = 0.28)	I: 0.71; S: 0.014 (p = 0.48)	0.84; 0.78–0.90 (p = 1.00)
Mid-costal	0.68; 0.66–0.72^#^ (p = 0.00)	0.75; 0.71–0.78	0.76; 0.72–0.77	0.77; 0.76–0.84	0.84; 0.77–0.86^§^ (p = 0.00)	I: 0.69; S: 0.036* (p = 0.00)	
Crural	0.79; 0.72–0.86 (p = 0.24)	0.80; 0.74–0.84	0.81; 0.69–0.84	0.83; 0.80–0.86	0.84; 0.80–0.85 (p = 1.00)	I: 0.79; S: 0.013 (p = 0.39)	

Day 1, 3, 9, 27, 81 and non-denervated side: Median values and interquartile ranges of normalized permutation entropy (PeEn). Day 1–81 regression: Intercept and slope of quantile regression model including 95% confidence intervals. ^#^: Significant difference between PeEn at day 1 vs. PeEn at non-denervated hemidiagphragm (corrected threshold p<0.05), ^§^: Significant difference between PeEn at day 1 vs. PeEn at day 81 (corrected threshold p<0.05). *: Significant slope of quantile regression between time after phrenicotomy and PeEn (corrected threshold p<0.05).

Additionally, PeEn was calculated at different settings, including low cut off frequencies *f_l_* between 0.5 Hz and 20 Hz, high cut off frequencies *f_h_* between 100 Hz and 400 Hz (adapted *f_s_* = 400 Hz to 1 kHz), dimensions *m* between 4 and 7, and time lags *l* between 1 and 5. Effects of denervation and consecutive reinnervation on PeEn remain consistent through the different settings at uncorrected threshold p<0.05 (PeEn sternal and mid-costal day 1 vs. non-denervated side; PeEn mid-costal day 1 vs. day 81).

## Discussion

This study demonstrates the successful implemention of PeEn, a method developed to provide reliable analysis of short and disturbed signals, to quantify denervation and reinnervation of the diaphragm.

Reinnervation of a muscle can be attributed to two mechanisms: outgrowth of a new motor axon back to its denervated muscle or terminal axon sprouting from adjacent intact motor units into the denervated fibers [25]. Removal of 20 mm of the phrenic nerve and cauterization of the proximal and distal nerve stump renders a direct reinnervation in this previously described model unlikely [7], [15]–[16]. Moreover, a vice versa collateral reinnervation of crural parts of the diaphragm via the main phrenic nerve has previously been described after resection of the accessory phrenic nerve [11]. Since reinnervation in this study occurred from crural towards sternal, it can be postulated that reinnervation rather originated from the accessory phrenic nerve, when present [7]. The presence of an accessory phrenic nerve innervating the crural parts of the diaphragm could be demonstrated in 76% of our rats. Since PeEn values in 10 of the 33 rats were significantly reduced in the crural part of the phrenicotomized hemidiaphragm, a possibility of collateral reinnervation can be excluded due to a missing dual innervation.

This study used an established model of unilateral phrenic nerve denervation, in which reinnervation has previously been confirmed by Gottschall et al. and Lullmann-Rauch [7], [15]. In addition we could provide histologic evidence of reinnervation in the form of fiber type grouping. However, none of these parameters are able to express the complex interactions across the neural and muscular network. The electromyogram measures comprises the intramuscular activity by detecting the compound motor action potential evoked by the accessory and main phrenic nerve. Thus the recorded electrical response (change in voltage) reflects a functional state of neuromuscular transmission. So far, electromyographic analysis is a clinically established method to indicate neuromuscular disorders [12] and represents a promising method to quantify the intramuscular effects of denervation and reinnervation. The diaphragm is a unique mammalian skeletal muscle with an equal proportion of slow and fast fibers providing highly resistance to fatigue and ensure continuous activity. Also unique is the necessity to adapt to various physiological conditions such as physical exercise [26]. Since the diaphragm is a skeletal muscle with arguably more than one nerval innervation, collateral reinnervation in case of muscular denervation is possible. Having the different muscle fiber composition in mind, these results can be transferred to at least a certain amount to all double innervated skeletal muscles. Quantitative analysis of the electromyographic signal can be performed by using different mathematical methods. Frequency analysis based on the Fourier spectrum e.g. provides a linear decomposition of the signal into its frequency content from which dominant frequencies can be identified. In clinical studies lower mean frequencies were usually detected in muscles of patients with neuropathies whereas in patients with myopathies, a shift towards higher elctromyographic frequencies has been reported [27]. Higher median frequencies in the diaphragmatic elctromyogram of elderly patients are associated with the recruitment of larger motor units [28]. However, changes of the frequency spectrum reflect only linear properties of the complex underlying neuromuscular dynamics. Furthermore, spectral analysis is sensitive to signal noise (e.g. electrocardiogram) and distortions (e.g. amplitude scales). Non-linear measures can detect additional information beyond linear randomness of the myographic activity which is related to the neuromuscular network structure. In electroencephalogram analysis, non-linear entropy plays a significant role in characterizing the complex interactions across the neuronal network by quantifying the order/disorder of a time series [18]–[20], [29]–[31]. In general, information theoretical approaches as used by entropy analysis indicate “information content” in a signal which is related to the amount of microstates in the underlying dynamic system. Approximate entropy could detect signal changes caused by progressive muscle fatigue and in the motor unit activity in patients with amyotrophic lateral sclerosis [32]–[35]. However, since approximate entropy underlies several limitations caused by the high dimensionality of signals and restricted analysis periods, non-parametric entropy measures were introduced representing an expedient approach to analyze physiological processes. We used PeEn as a robust measure of “signal complexity” in order to detect neural mechanisms of innervation, denervation and reinnervation. Therefore, PeEn values in the crural area of the diaphragm which were still innervated by the accessory phrenic nerve after phrenicotomy, remained within the normal range of the non-denervated hemidiaphragm. In contrast, PeEn in the mid-costal and sternal area was significantly decreased on day 1. As recruitment of larger motor units by a branching of the accessory phrenic nerve axons to the denervated muscle fibers progresses, the higher complexity of the neuromuscular network structure correlates with increasing entropy values. This can be demonstrated especially in the mid-costal area of the hemidiaphragm, where the significant increase of PeEn over time to normal values on day 81 demonstrates the electromyographic sign of gradual reinnervation. Although 8 of 10 rats clinically showed a contraction of the complete diaphragm 81 days after denervation, sternal PeEn could not detect a significant increase in values up to day 81 (Fig. 3A). This could be due to the fact that even though contractions were possible, the sternal area was not completely reinnervated; a fact which is consistent with our histological findings.

PeEn provides a robust estimate of denervation and reinnervation of the diaphragm as the obtained results were consistent over a broad interval of frequency bandwidth and embedding parameters. This observation is consistent with previous investigations using PeEn as a robust marker of consciousness and unconsciousness in electroencephalogram [19]–[20]. Therefore, PeEn seems to be suitable to reflect changes in physiological processes even if specific characteristics of the generating dynamic are still poorly understood (e.g. electroencephalogram, electromyogram).

### Limitations

The present analysis may be affected by background noise (mainly electrocardiogram) overlapping the electromyographic signals. Because recordings were performed successively on the specific areas a source separation of the electrocardiogram was not properly applicable. However, PeEn may show reliable results because of its robustness against amplitude outliers as given by electrocardiographic peaks. Furthermore, a statistical evaluation considering different parameters beside of PeEn to quantify denervation and reinnervation was not considered because of the relatively small sample size available. In the study PeEn represents a “proof of concept” to reliably indicate effects of denervation. Further investigations are required to identify electromyographic markers that are most suited to indicate denervation.

In summary, the application of PeEn on an electromyographic signal seems to be a new, distinctive measurement tool to characterize intramuscular processes following denervation and reinnervation.

## References

[1] Elefteriades J, Singh M, Tang P, Siegel MD, Kenney B, et al (2008) Unilateral diaphragm paralysis: etiology, impact, and natural history. J Cardiovasc Surg (Torino) 49:289–295. 18431352

[2] Kaufman MR, Elkwood AI, Rose MI, Patel T, Ashinoff R, et al (2011) Reinnervation of the paralyzed diaphragm: application of nerve surgery techniques following unilateral phrenic nerve injury. Chest 140:191–197. 21349932 10.1378/chest.10-2765

[3] Merino-Ramirez MA, Juan G, Ramon M, Cortijo J, Morcillo EJ (2007) Diaphragmatic paralysis following minor cervical trauma. Muscle Nerve 36:267–270. 17299741 10.1002/mus.20754

[4] Gibson GJ (1989) Diaphragmatic paresis: pathophysiology, clinical features, and investigation. Thorax 44:960–970. 2688182 10.1136/thx.44.11.960 PMC462156

[5] Douglass BE, Clagett OT (1960) The prognosis in idiopathic diaphragmatic paralysis. Dis Chest 37:294–297. 13817840

[6] de Vries TS, Koens BL, Vos A (1998) Surgical treatment of diaphragmatic eventration caused by phrenic nerve injury in the newborn. J Pediatr Surg 33:602–605. 9574760 10.1016/s0022-3468(98)90325-6

[7] Gottschall J, Gruber H (1977) The accessory phrenic nerve in the rat. Anat Embryol (Berl) 151:63–69. 907197 10.1007/BF00315298

[8] Marie JP, Tardif C, Lerosey Y, Gibon JF, Hellot MF, et al (1997) Selective resection of the phrenic nerve roots in rabbits. Part II: Respiratory effects. Respir Physiol 109:139–148. 9299645 10.1016/s0034-5687(97)00048-0

[9] Loukas M, Kinsella CR Jr, Louis RG Jr, Gandhi S, Curry B (2006) Surgical anatomy of the accessory phrenic nerve. Ann Thorac Surg 82:1870–1875. 17062263 10.1016/j.athoracsur.2006.05.098

[10] Nayak SR, Krishnamurthy A, Prabhu LV, Ramanathan L, Pai MM, et al (2008) Incidence of accessory phrenic nerve and its clinical significance: a cadaveric study. Acta Medica (Hradec Kralove) 51:181–184. 19271686 10.14712/18059694.2017.21

[11] De Bie G, Legrand A, Mahillon V, Lemort M, Gilles A, et al (2007) Schwannoma of the accessory phrenic nerve. Am J Otolaryngol 28:357–359. 17826542 10.1016/j.amjoto.2006.10.007

[12] Stevens RD, Marshall SA, Cornblath DR, Hoke A, Needham DM, et al (2009) A framework for diagnosing and classifying intensive care unit-acquired weakness. Crit Care Med 37:S299–308. 20046114 10.1097/CCM.0b013e3181b6ef67

[13] Ballantyne JP, Hansen S (1982) A quantitative assessment of reinnervation in the polyneuropathies. Muscle Nerve 5:S127–134. 6302489

[14] Iwasaki Y, Sugimoto H, Ikeda K, Takamiya K, Shiojima T, et al (1991) Muscle morphometry in amyotrophic lateral sclerosis. Int J Neurosci 58:165–170. 1365039 10.3109/00207459108985432

[15] Lullmann-Rauch R (1971) The regeneration of neuromuscular junctions during spontaneous re-innervation of the rat diaphragm. Z Zellforsch Mikrosk Anat 121:593–603. 5118528 10.1007/BF00560162

[16] Zhan WZ, Miyata H, Prakash YS, Sieck GC (1997) Metabolic and phenotypic adaptations of diaphragm muscle fibers with inactivation. J Appl Physiol 82:1145–1153. 9104851 10.1152/jappl.1997.82.4.1145

[17] Bandt C, Pompe B (2002) Permutation entropy: a natural complexity measure for time series. Phys Rev Lett 88:174102. 12005759 10.1103/PhysRevLett.88.174102

[18] Jordan D, Ilg R, Riedl V, Schorer A, Grimberg S, et al (2013) Simultaneous electroencephalographic and functional magnetic resonance imaging indicate impaired cortical top-down processing in association with anesthetic-induced unconsciousness. Anesthesiology 119:1031–1042. 23969561 10.1097/ALN.0b013e3182a7ca92

[19] Jordan D, Stockmanns G, Kochs EF, Pilge S, Schneider G (2008) Electroencephalographic order pattern analysis for the separation of consciousness and unconsciousness: an analysis of approximate entropy, permutation entropy, recurrence rate, and phase coupling of order recurrence plots. Anesthesiology 109:1014–1022. 19034098 10.1097/ALN.0b013e31818d6c55

[20] Olofsen E, Sleigh JW, Dahan A (2008) Permutation entropy of the electroencephalogram: a measure of anaesthetic drug effect. Br J Anaesth 101:810–821. 18852113 10.1093/bja/aen290

[21] Cao Y, Tung WW, Gao JB, Protopopescu VA, Hively LM (2004) Detecting dynamical changes in time series using the permutation entropy. Phys Rev E Stat Nonlin Soft Matter Phys 70:046217. 15600505 10.1103/PhysRevE.70.046217

[22] Kreuzer M, Kochs EF, Schneider G, Jordan D (2014) Non-stationarity of EEG during wakefulness and anaesthesia: advantages of EEG permutation entropy monitoring. J Clin Monit Comput. 10.1007/s10877-014-9553-y 24442330

[23] Brooke MH, Kaiser KK (1970) Muscle fiber types: how many and what kind? Arch Neurol 23:369–379. 4248905 10.1001/archneur.1970.00480280083010

[24] Goebel HH, Sewry CA, Weller RO (2013) Muscle Disease: Pathology and Genetics: Wiley-Blackwell. 392 p.

[25] Kugelberg E, Edstrom L, Abbruzzese M (1970) Mapping of motor units in experimentally reinnervated rat muscle. Interpretation of histochemical and atrophic fibre patterns in neurogenic lesions. J Neurol Neurosurg Psychiatry 33:319–329. 4246999 10.1136/jnnp.33.3.319 PMC493476

[26] Polla B, D'Antona G, Bottinelli R, Reggiani C (2004) Respiratory muscle fibres: specialisation and plasticity. Thorax 59:808–817. 15333861 10.1136/thx.2003.009894 PMC1747126

[27] Fuglsang-Frederiksen A, Ronager J (1990) EMG power spectrum, turns-amplitude analysis and motor unit potential duration in neuromuscular disorders. J Neurol Sci 97:81–91. 2370561 10.1016/0022-510x(90)90100-2

[28] Chen R, Collins SJ, Remtulla H, Parkes A, Bolton CF (1996) Needle EMG of the human diaphragm: power spectral analysis in normal subjects. Muscle Nerve 19:324–330. 8606696 10.1002/(SICI)1097-4598(199603)19:3<324::AID-MUS7>3.0.CO;2-F

[29] Jordan D, Ilg R, Schneider G, Stockmanns G, Kochs EF (2013) EEG Measures Indicating Anaesthesia Induced Changes of Cortical Information Processing. Biomed Tech (Berl). 10.1515/bmt-2013-4186 24042793

[30] Li D, Li X, Liang Z, Voss LJ, Sleigh JW (2010) Multiscale permutation entropy analysis of EEG recordings during sevoflurane anesthesia. J Neural Eng 7:046010. 20581428 10.1088/1741-2560/7/4/046010

[31] Thakor NV, Tong S (2004) Advances in quantitative electroencephalogram analysis methods. Annu Rev Biomed Eng 6:453–495. 15255777 10.1146/annurev.bioeng.5.040202.121601

[32] Zhou P, Barkhaus PE, Zhang X, Rymer WZ (2011) Characterizing the complexity of spontaneous motor unit patterns of amyotrophic lateral sclerosis using approximate entropy. J Neural Eng 8:066010. 22049095 10.1088/1741-2560/8/6/066010

[33] Harper NJ, Pugh ND, Healy TE, Petts HV (1987) Changes in the power spectrum of the evoked compound action potential of the adductor pollicis with the onset of neuromuscular blockade. Br J Anaesth 59:200–205. 2881566 10.1093/bja/59.2.200

[34] Hagg GM (1992) Interpretation of EMG spectral alterations and alteration indexes at sustained contraction. J Appl Physiol 73:1211–1217. 1447061 10.1152/jappl.1992.73.4.1211

[35] Xie HB, Guo JY, Zheng YP (2010) Fuzzy approximate entropy analysis of chaotic and natural complex systems: detecting muscle fatigue using electromyography signals. Ann Biomed Eng 38:1483–1496. 20099031 10.1007/s10439-010-9933-5

